# Factors that predict severity of infection and seroconversion in immunocompromised children and adolescents with COVID-19 infection

**DOI:** 10.3389/fimmu.2022.919762

**Published:** 2022-08-03

**Authors:** Mayada Abu Shanap, Maher Sughayer, Osama Alsmadi, Ismail Elzayat, Abeer Al-Nuirat, Abdelghani Tbakhi, Iyad Sultan

**Affiliations:** ^1^ Department of Pediatrics, King Hussein Cancer Center, Amman, Jordan; ^2^ Department of Pathology and Laboratory Medicine, King Hussein Cancer Center, Amman, Jordan; ^3^ Department of Cell Therapy and Applied Genomics, King Hussein Cancer Center, Amman, Jordan

**Keywords:** SARS-CoV-2, immunocompromised, COVID 19, cancer, children, stem cell transplant, seroconversion, chemotherapy

## Abstract

**Objectives:**

We aimed to study the outcomes, severity, and seroconversion post SARS-CoV-2 infection in immunocompromised children and adolescents treated at our center.

**Method:**

For this observational study, all pediatric patients who had COVID-19 infection from Sep-22-2020 to Nov-10-2021were identified by reviewing our laboratory records. Their charts were reviewed to determine clinical severity and outcome. Blood samples were drawn for anti-SARS-CoV-2 antibody assay. Serious COVID-19 infection (SVI) was defined if the patient had moderate, severe, or critical illness. A cutoff of 100 U/mL anti-SARS-CoV-2 antibodies was used to categorize low and high titer seroconversion.

**Results:**

We identified 263 pediatric patients with COVID-19; most (68%) were symptomatic: 5% had severe or critical infection, 25% were hospitalized, 12 required respiratory support, 12 were admitted to the ICU, and five patients (2%) died. Multivariable analysis revealed several factors that predict SVI: Age above 12 years (p=0.035), body mass index above 95^th^ percentile (p=0.034), comorbid conditions (p=0.025), absolute neutrophil count ≤500(p=0.014) and absolute lymphocyte count ≤300 (p=0.022). Levels of anti-SARS-CoV-2 spike antibodies were obtained for 173 patients at a median of 94 days (range, 14–300) after PCR diagnosis; of them 142 (82%) patients seroconverted; the lowest seroconversion rate was observed in patients with hematological malignancies (79%). Our univariable model showed that the following factors were predictive of low titer: lower ANC, p=0.01; hematologic malignancy, p=0.023; receiving steroids in the last 14 days, p=0.032; time since last chemotherapy or immunosuppressive therapy less than 30 days, p=0.002; and being on active chemotherapy in the last 3 months prior to infection, p<0.001.

**Conclusions:**

SARS-CoV-2 antibodies developed in most immunocompromised patients with COVID-19 infection in our study. Mortality was relatively low in our patients. Our univariable and multivariable models showed multiple variables that predict severity of infections and antibody response post COVID-19 infection. These observations may guide choice of active therapy during infection and the best timing of vaccination in this high-risk population.

## Introduction

Although most COVID-19 infections are mild/asymptomatic, several observational cohorts have identified that being older, male, and having multiple comorbid conditions, including obesity, diabetes (especially with elevated hemoglobin A1c), severe asthma, respiratory or heart disease, autoimmune and immunosuppressive conditions, renal failure, and hematologic malignancy or other cancers, are predictors of severe COVID-19 infection and mortality (1, 2). Pediatric patients with primary and secondary immunodeficiency diagnosed with COVID-19 infection have variable outcome, though most (85-90%) survive the illness; they are at higher risk of severe illness and death compared with the general pediatric population; this risk may be lower than that observed in their adult oncology counterparts (3).

While antibody and T cell responses to SARS-CoV-2’s structural proteins in healthy convalescent donors are well described, adaptive humoral and cellular immunity has not yet been characterized in pediatric immunocompromised patients (4–8). Preliminary studies in non-immunocompromised subjects with COVID-19 infection reported seroconversion 7 to 14 days following symptom onset, with increased IgM and IgG titers observed during the first month (9–13). Antibody responses among immunocompromised patients, including patients with cancer and hematopoietic stem-cell transplant recipients infected with SARS-CoV-2, may be diminished compared to those of the general population and have not been fully characterized.

It is important to understand the quality of the immune response post natural COVID-19 infection in this highly vulnerable immune-compromised population. Such knowledge will help to predict how effective COVID-19 vaccines are in immunocompromised individuals and which patients will benefit or mount a protective immune response. We aimed to evaluate disease severity and antibody response among our patients prior to vaccination. We identified patients who were infected during the first COVID-19 outbreak in Jordan, which peaked in the middle of November 2020.

## Patients and methods

### Study design and participants

This is an observational study that was approved by our Institutional Review Board (IRB) (study# 20 KHCC 192 F) for children and adolescent (<19 years at diagnosis) with COVID-19 infection who were treated at our center. Inclusion criteria included having a current or past diagnosis of cancer, having received a hematopoietic stem-cell transplantation, or having benign hematologic disorder or primary immune deficiency (PID).

COVID-19 infection was confirmed in all patients by SARS-CoV-2 reverse transcription-polymerase chain reaction (RT-PCR) assay from an oropharyngeal or nasopharyngeal swab collected when they had symptoms suggestive of COVID-19, or swabs from asymptomatic patients who had COVID-19–infected contacts, or surveillance swabs collected before anesthesia or hospital admission.

After consenting, patients (or their parents) were asked to complete a questionnaire about their exposure, health status, and symptoms. Serology testing was drawn 14 days or more after diagnosis. We collected data on demographic variables (age, sex, diagnosis, body mass index), comorbidities (excluding cancer itself), SARS-CoV-2 spike antibody result, SARS-CoV-2 RT–PCR result, cancer treatment history, onset of symptoms of COVID-19, subsequent disease course, treatment setting, outcomes, date of infection, and date of transplant. Laboratory data, including absolute lymphocyte count and absolute neutrophil count, were reviewed.

### SARS-CoV-2 antibody testing

Anti-SARS-CoV-2 spike protein antibodies (Abs) were tested by using the Roche SARS-COV-2 Abs assay (The Elecsys Anti-SARS-CoV-2 S immunoassay) This assay is a quantitative electro-chemiluminescent immune-assay (ECLIA) that detects high-affinity antibodies to the SARS-CoV-2 S protein’s RBD and has a low risk of detecting weakly cross-reactive and unspecific antibodies. Results are automatically reported as the analyte concentration of each sample in U/mL, with <0.80 U/mL interpreted as being negative for anti-SARS-CoV-2 S antibodies, and ≥0.80 U/mL interpreted as being positive for anti-SARS-CoV-2 S antibodies (Roche Diagnostics GmbH. Elecsys Anti-SARS-CoV-2 S assay method sheet. 2020; version 2.0) (Roche Diagnostics GmbH, 2021c) (14).

We considered an antibody titer of 100 as a cutoff value for the differentiation between weak and strong serological responses (median absolute antibody level=100 U/mL, low Abs titer ≤100 U/mL, high Abs titer >100 U/mL).

### Statistical analysis

Patients’ characteristics were analyzed and reported by using descriptive statistics. Continuous variables are expressed as median and range; categorical variables are presented as frequencies and percentages. We calculated the seroconversion rate as the proportion of confirmed reactive to total specimen. Categorical variables were compared by chi-squared testing. Continuous variables were evaluated as means with standard deviations (+/-SD) and were compared by Student’s t-testing. A p-value of <.05 was considered to be statistically significant. For smaller subsets of patients, continuous variables were represented as medians with interquartile range (IQR).

Univariable and multivariable logistic regression models were applied to study the association between severity of infections and the following variables: age, sex, comorbidities, primary disease, COVID-19 disease severity, hospitalization, last therapy applied and time to COVID-19, active therapy (time from last therapy to COVID-19 within three months), and time from COVID-19 to first serology evaluation.

A univariable logistic regression model was used to assess the association between antibody titer and baseline characteristics such as age, sex, cancer type, ALC, ANC, status of therapy, and type of last therapy. All statistical analyses were done with R statistical software version (4.0.2).

## Results

### Patient selection

**Figure 1:**
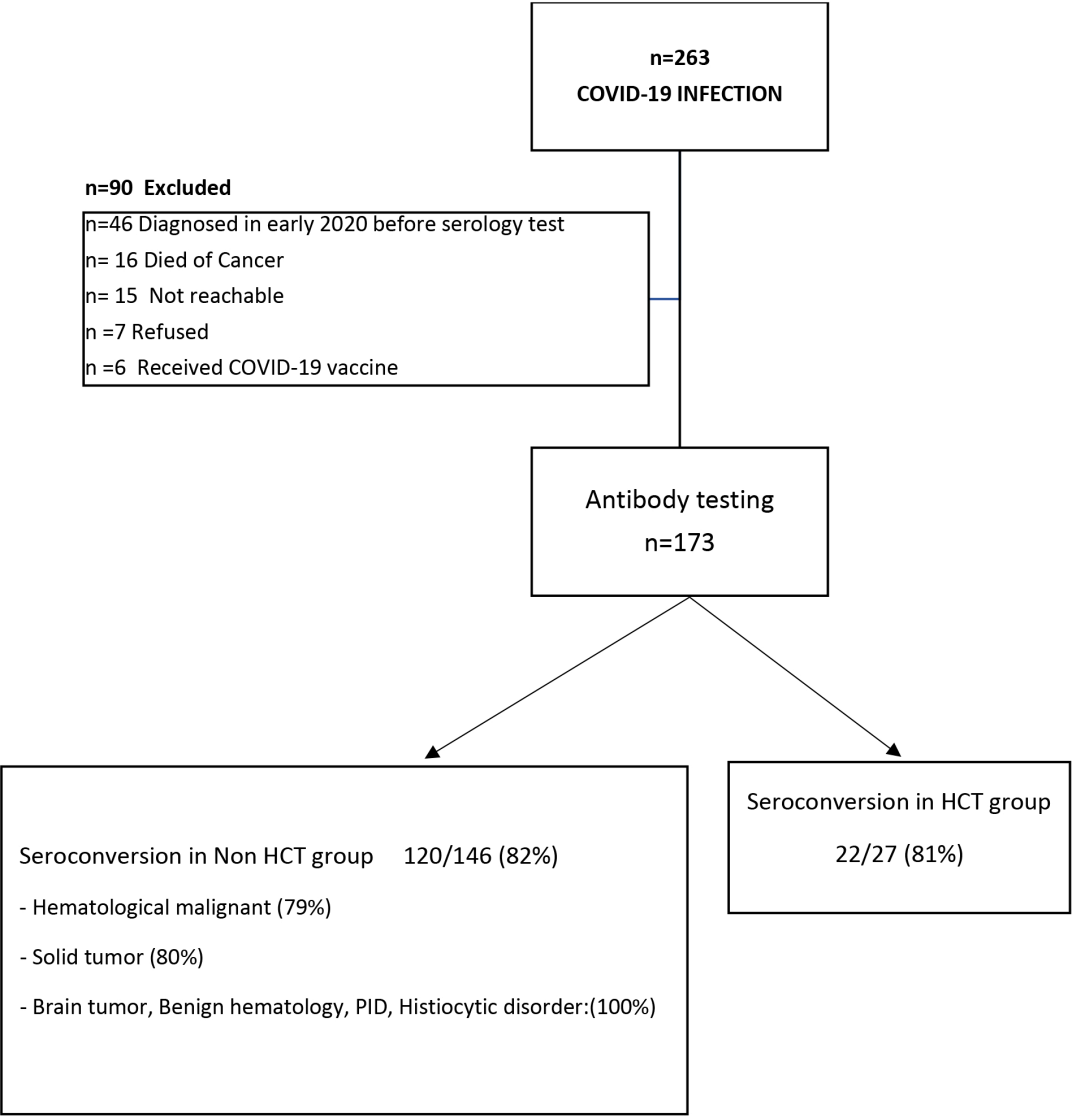
Cohort selection and Seroconversion rate post COVID-19 infection: A total of 263 patients with COVID-19 infection were included in this study; of them 173 patients (65%) underwent serology testing, while 90 patients were excluded. Of the 173 patients tested, 142 patients (82%) seroconverted/had a positive Anti-SARS-CoV-2 S antibody; seroconversion rate in patients underwent hematopoietic stem cell transplant (HCT) is 81%, while in non-HCT group is 82%.

A total of 263 patients were included in this study (female, n=110, 42%). The mean age of included individuals was 10.8 years (+/-6.76 y, range 0.73-31.3 y); 25 patients (9.5%) were more than 20-years and included as they were followed by our pediatric service. We identified all patients with COVID-19 infection and having a diagnosis of either cancer (n=204, 77.6%), benign hematologic and histiocytic disorders (n=12, 4.5%), PID/not transplanted (n=2, 0.8%), patients who received hematopoietic stem-cell transplantation (HCT, n= 44, 16.7%) and one patient with rheumatologic disorder (0.4%) (**Table 1A**). Most patients (n=173, 66%) were identified for serology testing; while (n=90,34%) patients were excluded for the following reasons: n=46 diagnosed with COVID-19 infection in early 2020 before serology testing was available at our center, n= 16 died of cancer before testing, n= 15 were not reachable, n=7 refused, and n=6 received a COVID-19 vaccine (**Figure 1**).

**Table 1A:** Baseline characteristics of patients with COVID-19 infection*.

Variables	n (%)
**Age** < 12 ≥12 Median (range) Mean (SD)	159 (60%) 104 (40%) 9.4 y (0.73-31) 10.8 y ± (6.8)
**Sex** Male Female	153 (58%) 110 (42%)
**Type of Cancer** Hematological malignancy Solid tumor Brain tumor Post HCT (malignant) Post HCT (nonmalignant) Other**	136 (52%) 45 (17%) 23 (9%) 20 (7%) 24 (9%) 15 (6%)
**Type of cancer-directed therapy** Chemotherapy Chemotherapy+anti-CD20 Targeted therapy/TKI HCT HCT+ Anti-PD1 No chemotherapy	185 (70%) 5 (2%) 4 (1.5%) 42 (16%) 2 (1%) 25 (9.5%)
**Time since transplantation** 31-99 days 100 -300 days >300 days	6/44 (14%) 7 (16%) 31 (70%)
**Steroids in last 14 days** Yes No	42 (16%) 221 (84%)
**Intense therapy** Yes No	60 (23%) 203 (77%)
**Active/ongoing therapy** Yes No	198 (75%) 65 (25%)
**Time since last chemo/IS** <30 ≥30 No chemotherapy	193 (73%) 45 (17%) 25 (10%)
**Radiation** Yes No	58 (22%) 205 (78%)
**No. of comorbidities** 0 1 ≥2	172 (65%) 81 (31%) 10 (4%)
**BMI** <95^th^ percentile ≥95^th^ percentile	243 (92%) 20 (8%)
**ANC** ≤500 >500	77 (29%) 186 (71%)
**ALC** ≤300 >300	42 (16%) 221 (84%)

*n=263 patients with COVID-19 infection

** Other: Inherited bone marrow failure syndrome (IBMF), Idiopathic severe aplastic anemia (SAA), hemoglobinopathies (Thalassemia, sickle cell disease, Hereditary spherocytosis), Histiocytic disorder (LCH), systemic juvenile idiopathic arthritis

standard deviation (SD).

ALC, absolute lymphocyte count; ANC, absolute neutrophil count; BMI, body mass index; COVID‐19, coronavirus disease 2019; HCT, hematopoietic stem cell transplant; TKI, tyrosine kinase inhibitors; IS, immune-suppressive therapy.

Among the 204 patients with cancer, 136 had a hematological malignancy; 45 had a solid tumor, and 23 had a brain tumor. Almost three-quarters (n=193) had received chemotherapy/immune-suppressive (IS) therapy in the last month prior to COVID-19 infection; 60 of them had received intensive therapy. Forty-two had received steroid therapy in the last 14 days prior to COVID-19 infection. Of the HCT recipients (n=44), only 6 had infections within 100 days post-transplant. Seven patients had received radiation therapy within 30 days of COVID-19 infection. Comorbidities were documented in 91 patients (**Table 1A**).

### Diagnosis and clinical course of COVID‐19 infection

**Figure 2:**
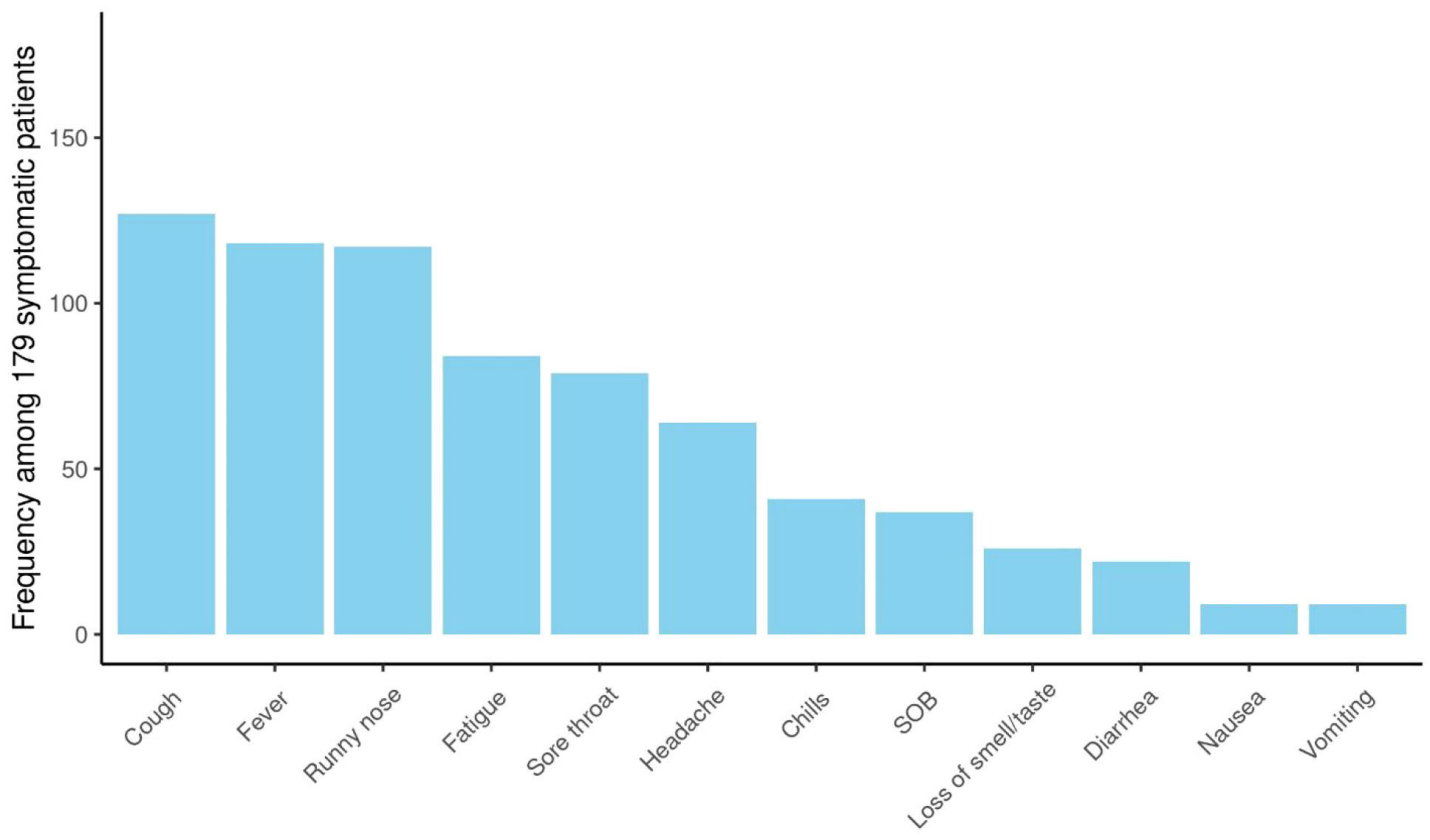
Symptomatology of COVID-19 infection: Among patients with COVID-19 infection (n= 263); 179 patients were symptomatic. The most commonly reported symptoms at presentation were cough (n=127, 71%), fever (n=118, 66%), runny nose (n=117, 65%).

**Figure 3:**
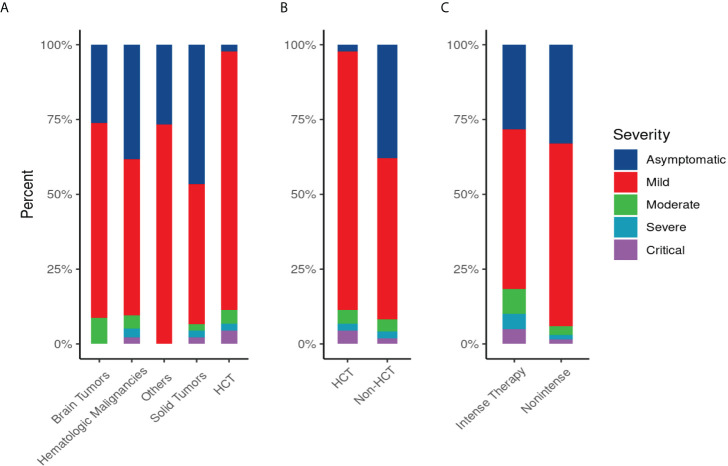
Disease Severity Score of COVID-19 infection by primary diagnosis **(A)**, by type of therapy “hematopoietic stem cell transplant (HCT) vs non HCT” **(B)**, and by intensity of therapy**(C)**: **(A)** Severity of COVID-19 infection by primary diagnosis: SVI was common in patients with hematological malignancy (13/136;10%), in patients who underwent HCT (5/44; 11%), followed by brain tumor (2/23; 8.6%) and solid tumor (3/45;6.6%). **(B)**Severity of COVID-19 infection in hematopoietic stem cell transplant (HCT) or non-HCT group: SVI in HCT (5/44; 11%) versus (18/219; 8%) in non-HCT group. **(C)** Severity of COVID-19 infection by intensity of therapy: SVI was common in patients who received intense therapy (11/60; 18%) versus (12/203; 6%) in patients received non-intense therapy.

More than two-thirds had symptomatic infections (n=179), and 84 patients (32%) were asymptomatic. The most commonly reported symptoms at presentation were cough (n=127, 71%) and fever (n=118, 66%) (**Figure 2**). Most symptomatic patients had mild COVID-19 infection (n=156, 87%); while serious COVID-19 infection (SVI) was reported in 23 patients: Moderate (n=11, 6%), severe/critical infections (n=12, 7%) (**Table 1B**) (**Figure 3**). Fifty-three (20%) of 263 patients required admission to the floor; and additional (n=12) to the intensive care unit (ICU); half of these (n=6) required mechanical ventilation. Thirty-eight of 179 patients (21%) met the definition of febrile neutropenia at admission (**Table 1B**). Twenty‐one patients received COVID-19–directed therapy; steroids, IVIG, and azithromycin were used frequently (18 of 21), and tocilizumab was used in five patients. Anticoagulation was started in 12 hospitalized patients as a prophylaxis in patients with severe to critical disease. There were 25 (9.5%) deaths in this cohort; five of them (20%) were attributed to COVID-19 infection. Median time to death attributed to COVID-19 was 21 days (range, 9-30 days) (**Table 1B**). Multiple factors were predictive of the severity of infection in our multivariate model: Age above 12 years (OR, 3.4;95%CI,1.1-10.9; p=0.035), BMI≥95th percentile (OR, 5; 95%CI,1.2-21; p=0.034), patients with comorbid conditions (OR, 3; 95%CI,1.2-8.3; p=0.025), ANC ≤500 (OR, 4.34; 95% CI, 1.38-14.32, p=0.014), and ALC ≤ 300 (OR, 4; 95% CI, 1.2-14.2; p=0.022) (**Table 2**). Of note, the type of cancer did not affect severity in either univariable or multivariable models.

**Table 1B:** Outcomes of Patients with COVID-19 infection*.

Variables	n (%)	
**COVID-19 Severity** Asymptomatic Mild Moderate Severe Critical	84 (32%) 156 (59%) 11 (4%) 6 (2.5%) 6 (2.5%)	
**Resolution of infection** Yes No	258 (98%) 5 (2%)	
**Hospitalization** Yes/floor Yes/ICU No	53 (20%) 12 (5%) 198 (75%)	
**COVID-19–directed therapy** Yes No	21 (8%) 242 (92%)	
**COVID-19 mortality** Yes No	5 (2%) 258 (98%)	
**Seroconversion** Yes No	142/173 (82%) 31/173 (18%)	
**Titer** >100 0.8-100 <0.8	72/173 (42%) 70/173 (40%) 31/173 (18%)	

*n=263, patients COVID‐19, coronavirus disease 2019.

**Table 2:** Univariable and multivariable analysis for disease severity (n=263 patients).

Variables	Asymptomatic/Mildn=240	Moderate/Severe/Criticaln=23	Univariable Analysis p value OR (95% CI)		Multivariable analysis p value OR (95% CI)	
**Age** <12 >=12	152 (95.6%) 88 (84.6%)	7 (4.4%) 16 (15.4%)	**0.004**	1 (ref) 4 (1.6-10.6)	**0.035**	1 (ref) 3.4 (1.1-10.9)
**Sex** Male Female	136 (88.9%) 104 (94.5%)	17 (11.1%) 6 (5.5%)	0.116	2 (0.8-6.2) 1 (ref)	–	
**Type of Cancer** Hem/malignancy Solid Tumor Brain Tumor Other	123 (90.4%) 42 (93.3%) 21 (91.3%) 54 (91.5%)	13 (9.6%) 3 (6.7%) 2 (8.7%) 5 (8.5%)	0.896 0.762 0.974	1 (0.3-7.4) 0.8 (0.1-6) 1 (ref) 0.9 (0.2-7)	–	
**Type of Therapy** HCT Non HCT	39 (88.6%) 201 (91.8%)	5 (11.4%) 18 (8.2%)	0.50	1 (ref) 0.7 (0.3-2)	–	
**Steroid in last 14 days** Yes No	35 (83.3%) 205 (92.7%)	7 (16.7%) 16 (7.2%)	0.05	2.6 (0.9-6.5) 1 (ref)	–	
**Intense Therapy** Yes No	49 (81.7%) 191 (94.1%)	11 (18.3%) 12 (5.9%)	**0.004**	3.6 (1.5-8.6) 1 (ref)	0.81	1.2 (0.3-4) 1 (ref)
**Active/Ongoing Therapy** Yes No	179 (90.4%) 61 (93.8%)	19 (9.6%) 4 (6.2%)	0.39	1.6 (0.6-5.8) 1 (ref)	–	
**Number of Comorbidities** 0 >=1	167 (97.1%) 73 (80.2%)	5 (2.9%) 18 (19.8%)	**<0.001**	1 (ref) 4.4 (2.3-9.9)	**0.025**	1 (ref) 3 (1.2-8.3)
**Time since last chemo/IS** <30 ≥30	41 (91.1%) 174 (90.2%)	4 (8.9%) 19 (9.8%)	0.85	1 (ref) 0.9 (0.3-2.5)		
**BMI** <95^th^ percentile >=95^th^ percentile	228 (93.8%) 12 (60%)	15 (6.2%) 8 (40%)	**<0.001**	1 (ref) 10 (3.5-28)	**0.034**	1 (ref) 5 (1.2-21)
**ALC** ≤300 >300	32 (76.2%) 208 (94.1%)	10 (23.8%) 13 (5.9%)	**<0.001**	1 (ref) 0.2 (0.1-0.5)	**0.022**	1 (ref) 4 (1.2-14)
**ANC** ≤500 >500	63 (81.8%) 177 (95.2%)	14 (18.2%) 9 (4.8%)	**0.001**	1 (ref) 0.2 (0.1-0.6)	**0.014**	1 (ref) 4.3 (1.4-14)

ALC, absolute lymphocyte count; ANC, absolute neutrophil count; BMI, body mass index; COVID‐19, coronavirus disease 2019; HCT, hematopoietic stem cell transplant; IS, immune-suppressive therapy. Bold means to highlights significant p value.

### Anti-SARS-CoV-2 spike antibody serology

Of the 173 patients tested, 142 (82%) had a positive Anti-SARS-CoV-2 S antibody test, the remainder had a negative SARS-CoV-2 S antibody test (**Table 1B**). The median time between SARS-CoV-2 RT-PCR and SARS-CoV-2 S antibody testing was 94 days (range, 14-300). Days to testing did not significantly differ between patients who were seronegative (median, 104; range, 14-232) and those who were seropositive (median, 94; range 14-300). Seroconversion rate in patients with hematological malignancies was (79%), HCT patients (81%), and solid tumors (80%). Notably, all patients with brain tumors, benign hematology/PID/Histiocytic disorder developed SARS-CoV-2 S antibody (100% seroconversion rate).

Among patients who had a positive antibody test (N=142), 72 had a high titer (>100 U/mL) and 70 had a low titer (0.8 to 100 U/mL) (**Table 1B**). Patients with hematologic malignancies were less likely to have a high titer; and this finding was positive in our univariable model (OR, 0.15; 95%CI, 0.02-0.66; p=0.023) but not significant in our multivariable model (OR, 0.09, 95%CI, 0.00-0.8; p=0.064) (**Table 3**). Our univariable model showed the following factors to be predictive of low titer: lower ANC (p=0. 0.01); receiving steroids in the last 14 days (p=0.023); time since last chemotherapy or immunosuppressive therapy less than 30 days (p=0.002); and being on active chemotherapy in the last 3 months(p<0.001); and non-HCT group (p=0.03). None of these factors were significant predictors of high titer in our multivariable model.

**Table 3:** Univariable and multivariable analysis for seroconversion (n=142 patients).

Variables	Low Titer (0.8 - 100 U/ml)n=70	High titer (>100 U/ml)n=72	Univariable Analysis		Multivariable analysis		
			p value	OR (95% CI)	p value	OR (95% CI)
**Age** <12 >=12	49 (53.8%) 21 (41.2%)	42 (46.2%) 30 (58.8%)	0.15	1 (ref) 1.7 (0.8-3.4)		
**Sex** Male Female	39 (48.8%) 31 (50%)	41 (51.2%) 31 (50%)	0.88	1.1(0.5-2) 1 (ref)		
**Type of Cancer** Hematological malignancy Solid Tumor Brain Tumor Other	52 (61.9%) 7 (43.8%) 2 (20%) 9 (28.1%)	32 (38.1%) 9 (56.2%) 8 (80%) 23 (71.9%)	**0.023**	0.15 (0.02-0.7) 0.32 (0.04-1.8) 1 (ref) 0.64 (0.09-3.2)	**0.064**	0.09 (0.00-0.8) 0.19 (0.01-1.9) 1 (ref) 0.18 (0.01-2.9)
**Type of Therapy** HCT Non HCT	6 (27.3%) 64 (53.3%)	16 (72.7%) 56 (46.7%)	**0.030**	1 (ref) 0.33 (0.11-0.9)	0.42	1 (ref) 0.37 (0.03-4.1)
**Steroids in last 14 days** Yes No	19 (67.9%) 51 (44.7%)	9 (32.1%) 63 (55.3%)	**0.032**	0.38 (0.15-0.9) 1 (ref)	0.39	0.65 (0.23-1.7) 1 (ref)
**Intense Therapy** Yes No	20 (54.1%) 50 (47.6%)	17 (45.9%) 55 (52.4%)	0.5	0.77(0.36-1.6) 1 (ref)		
**Active/Ongoing Therapy** Yes No	67 (57.8%) 3 (11.5%)	49 (42.2%) 23 (88.5%)	**<0.001**	0.10 (0.02-0.3) 1 (ref)	0.55	0.33 (0.01-17) 1 (ref)
**Time since last chemo/IS** <30 ≥30	66 (57.9%) 3 (15.8%)	48 (42.1%) 16 (84.2%)	**0.002**	1 (ref) 7.3 (2.3-32)	0.76	1 (ref) 1.7(0.04-67)
**Radiation** Yes No	9 (36%) 61 (52.1%)	16 (64%) 56 (47.9%)	0.147	1.94 (0.81-4.9) 1 (ref)	0.30	0.39 (0.05-2.2) 1 (ref)
**ANC** ≤500 >500	31 (64.6%) 39 (41.5%)	17 (35.4%) 55 (58.5%)	**0.01**	1 (ref) 2.57 (1.3-5.4)	0.15	1 (ref) 1.83 (0.81-4.2)
**ALC** ≤300 >300	14 (58.3%) 56 (47.5%)	10 (41.7%) 62 (52.5%)	0.24	1 (ref) 1.55 (0.64-3.7)		

ALC, absolute lymphocyte count; ANC, absolute neutrophil count; BMI, body mass index; COVID‐19, coronavirus disease 2019; HCT, hematopoietic stem cell transplant; IS, immune-suppressive therapy. Bold means to highlights significant p value.

## Discussion

We analyzed the clinical and laboratory parameters of 263 patients with secondary immunodeficiency and confirmed SARS-CoV-2 infection; two-thirds of our patients (68%) were symptomatic; 5% developed severe or critical COVID-19–related illness, and 2% died due to SARS-CoV-2 infection. Mortality and severity were lower than what is reported in adults with cancer; however, disease severity was worse than that in the general pediatric population (3–8, 15).

The Pediatric Oncology COVID-19 Case Report registry is an observational study that followed children with cancer treated at 94 institutions. They reported findings in 917 patients with cancer and COVID-19. Similar to our results, 64% were symptomatic, 10.9% required respiratory support, and 1.6% died. Hispanic ethnicity was over-represented in this sample and was more likely to be associated with changes in cancer-directed therapy (16). A larger proportion of severe or critical illness in low- and middle-income countries compared with case series from high-income countries reported by St Jude Global and International Society of Pediatric Oncology Global Registry of COVID-19 in Childhood Cancer (GRCCC) (17). The American Society of Hematology (ASH) registry reported a pediatric mortality rate of 3% as of December 10, 2021 (15).

Similar to the literature, we identified multiple factors that were significantly predictive of disease severity in a multivariable model: age, BMI, intensive therapy, ANC, ALC, and having comorbidities. Interestingly, type of cancer did not predict disease severity.

Lymphopenia has been described to correlate with more severe COVID-19 illness in both immunocompetent and immunocompromised patient populations. Even in the early stages of infection, lymphopenia is commonly seen in patients with COVID-19 (18, 19). In a recently published meta-analysis, patients with severe COVID-19 had lower lymphocyte counts than did those with non-severe COVID-19 (20). Also, it can be used as predictive markers of increased risk of in-hospital mortality and ICU admission (20). Lymphopenia post COVID-19 infection can be explained by four factors. First, SARS-CoV-2 might directly infect and destroy splenic and lymph nodal follicles, as indicated by the post mortem analysis of 6 patients previously reported by Feng et al. (21). Second, activated lymphocytes might be sequestered in the injured tissues, especially in the lung (22).Third, the production of lymphocyte precursors by the bone marrow and the thymus might be exhausted (23). Fourth, it is chemotherapy-induced functional exhaustion of antiviral lymphocytes.

Data regarding antibody responses after COVID-19 infection in immunocompromised pediatric patients are limited. In our cohort, 82% of patients manifested a positive anti-spike antibody response, which appears to closely correlate with the neutralizing capacity (24). Most patients included in our study were tested for antibody response more than 6 weeks after COVID-19 infection [median, 14 weeks; range, 2-42 weeks]. In our cohort, 54 asymptomatic patients were tested for COVID-19 antibodies. Of these, 39 (72%) had detectable SARS-CoV-2 antibodies, suggesting that asymptomatic infection also leads to seroconversion in most cases and may contribute to expansion of the pandemic and herd immunity.

Variable rates and patterns of seroconversion have been described in adult patients with cancer. Astha Thakkar et al. reported significantly lower seroconversion in patients with hematologic malignancies (82%), patients who received anti-CD-20 antibody therapy (59%), and patents who received stem cell transplant (60%); yet, SARS-Cov-2 IgG antibodies developed in patients who received immunotherapy, including those who received anti-PD-1/PD-L1 monoclonal antibodies (100% seroconversion) (25). Anna Candoni reported a seroconversion rate of 84% in patients with hematological malignancies; however, the kinetics of the antibody levels suggest that the duration of antibody‐mediated protection against re‐infection with SARS-CoV‐ 2 may be short‐lived (26). Additionally, in a patient series of chronic lymphocytic leukemia with COVID-19 infection, fourteen of 21 (67%) patients tested positive for anti-SARS-CoV-2 Abs (27).

The ITA-HEMA-COV project (NCT04352556) investigated patterns of seroconversion for SARS-CoV-2 IgG in patients with hematological malignancy. Overall, 69% of patients had detectable IgG SARS-CoV-2 serum antibodies. In the multivariable logistic regression, chemoimmunotherapy was associated with a lower rate of seroconversion (28). In our study, we observed clinically and statistically significant differences in seroconversion (low antibody response) in patients who had received recent chemotherapy/IS. We noted strong trends toward inferior seropositivity rates among patients receiving steroid therapy before SARS-CoV-2 infection and in those who were treated for hematological malignancy or were neutropenic at the peak of infection. In multivariable analysis, hematologic cancer remained the only factor associated with low antibody response, as these malignancies directly involve the lymphoid and myeloid immune compartments and their therapy depletes B-cells in lymphoid tissues and bone marrow, which would alter the antibody response post viral infection.

The low antibody response post COVID-19 infection in pediatric patients with hematologic cancer was not associated with increased mortality compared to that of COVID-19–infected pediatric patients with other cancers; this observation warrants studying T cells’ immune response post COVID-19 infection in this unique population of immunocompromised patients. In two cohorts of adults with hematologic malignancy at University of Pennsylvania and Memorial Sloan Kettering Cancer Center, higher CD8+ T cell counts were associated with improved overall survival. CD8+ T cells likely compensate for deficient humoral immunity and influence clinical recovery of COVID-19. This study highlights the importance of CD8+ T cells in acute COVID-19, particularly in the setting of impaired humoral immunity (29).

Data regarding the immune response post primary and secondary immunodeficiencies in children are still scarce. Case reports of the immune response to natural SARS-SoV-2 infection in those with primary immunodeficiency suggest that such patients can develop both humoral and cell-mediated immune responses to the virus (30). In addition, a small study of patients with primary immunodeficiencies found that 69% were seropositive and 73% mounted a SARS-CoV-2-specific T cell response after vaccination (31).

Another study reported dynamics of the anti-SARS-CoV-2 antibody response; seroconversion was observed in 92% of patients (16 with ALL, and 2 brain tumors) by week 3 and in 100% of patients by week 6 post exposure. The seropositivity rate was maintained at around 80% for at least three consecutive weeks, declining to 54% by week 18 post exposure (32).

Forty-four patients in our study were recipients of HCT; most of them had mild COVID-19 infection, with a high rate of seroconversion (81%). These findings are likely explained by the facts that most HCT recipients in our cohort were >300 days post-transplant, half of them were off IS, and most did not have GVHD (75%). Previous reports showed that most HCT recipients can mount an immune response after infection and that lymphocyte count was predictive of antibody titer (33, 34).

Several studies have shown a reduced immunologic response to COVID-19 vaccination among people with various immunocompromising conditions. Compared with those who are not immunocompromised, specific groups of immunocompromised adults including people receiving solid organ transplants (35–37); people with cancer, particularly hematologic cancers (38, 39), and people receiving hemodialysis for kidney disease (40, 41) have shown reduced antibody response to two doses of mRNA vaccines.

A study of more than 500 patients with cancer mirrored these trends: those with solid tumors had a much higher rate of seropositivity (93%) than did those with hematological cancers (66%) (42).In another report, failure to mount an immune response correlated significantly with steroid treatment (43)

Our study suffers from some limitations. First, no assays of cellular immunity were done. Timing of antibody testing was not standardized, varying depending on testing availability and the timing of patients’ visits. We did not follow patients longitudinally to study the kinetics of serologic conversion and rates of reinfection. It is possible, for example, that some seronegative patients had early but nondurable SARS-CoV-2 IgG responses; we cannot exclude the possibility that these patients had transient immune responses not captured by the testing window. Nevertheless, we did not observe significant changes in titer levels according to the time of sampling.

Vaccination against SARS-CoV-2 might be the most promising approach to durably stop the current COVID-19 pandemic; these data have important implications for understanding the immune response in immunocompromised pediatric patients post COVID-19 infection. Such knowledge can be used to guide the timing of vaccination in patients receiving chemotherapy and immunosuppressive agents as well as to determine the need for them to get booster doses of vaccine. The current vaccines available for COVID-19 are not live vaccines and should be safe for use in immunocompromised children and adolescents, but the efficacy will have to be followed carefully for each immune defect.

## Data availability statement

The original contributions presented in the study are included in the article/supplementary material. Further inquiries can be directed to the corresponding author.

## Ethics statement

The studies involving human participants were reviewed and approved by Institutional Review Board (IRB) at King Hussein Cancer Center (study# 20 KHCC 192 F). Written informed consent to participate in this study was provided by the participants’ legal guardian/next of kin.

## Author contributions

MA, contributed to conceptualization and methodology of the study, interpreted data, and wrote the manuscript. IS, statistical analysis and edited the manuscript. MS, supervised the serology testing and edited the manuscript. AT and OA, molecular testing and edited the manuscript. IA, consenting patients and data collection. AA-N, performed the serology testing. All authors contributed to the article and approved the submitted version.

## Acknowledgments

We acknowledge KHCC intramural grant (# 20 KHCC 192 F) in providing funding for this project. The funder had no role in study design, data collection and analysis, decision to publish or preparation of the manuscript.

We thank Cherise Guess, the senior scientific editor at St. Jude Children’s Research Hospital who contributed to scientific editing of the manuscript.

## Conflict of interest

The authors declare that the research was conducted in the absence of any commercial or financial relationships that could be construed as a potential conflict of interest.

## Publisher’s note

All claims expressed in this article are solely those of the authors and do not necessarily represent those of their affiliated organizations, or those of the publisher, the editors and the reviewers. Any product that may be evaluated in this article, or claim that may be made by its manufacturer, is not guaranteed or endorsed by the publisher.

## Abbreviations

Ab, antibody; ALC, absolute lymphocyte count; ANC, absolute neutrophil count; ALL, acute lymphoblastic leukemia; AML, acute myeloid leukemia; BMI, body mass index; COVID‐19, coronavirus disease 2019; ECLIA, electro-chemiluminescent immune-assay; HCT, hematopoietic stem cell transplant; ICU, intensive care unit; IS, immune-suppressive therapy; GVHD, graft versus host Disease; PCR, polymerase chain reaction; PID, primary immune deficiency; SARS‐CoV‐2, severe acute respiratory syndrome coronavirus‐2; SARS-CoV-2 S, severe acute respiratory syndrome coronavirus‐2 spike protein; SOB, shortness of breath; SVI, serious COVID-19 infection; RT-PCR, reverse transcription-polymerase chain reaction.
